# An Integrated Methodological Framework for SoilHealth Assessment in Volcanic Island Agroecosystems: Indicator Selection, Analytical Workflows and Context-Sensitive Interpretation

**DOI:** 10.3390/mps9050129

**Published:** 2026-09-01

**Authors:** Sara S. González-González, Mónica González-González

**Affiliations:** Instituto Canario de Investigaciones Agrarias (ICIA), 38270 San Cristóbal de La Laguna, Tenerife, Spain; mgonzalez@icia.es

**Keywords:** soil health, volcanic soils, Andosols, island agroecosystems, soil indicators, analytical workflows, method suitability, context-sensitive interpretation, volcanic ash

## Abstract

Soil health assessment is increasingly used to support sustainable land management and agronomic decision-making, but generic indicator lists and universal scoring systems may be inadequate for highly reactive volcanic soils. This critical methodological review proposes an integrated methodological framework for volcanic island agroecosystems that explicitly separates analytical measurement from interpretation. The framework links assessment objectives and site context to fit-for-purpose analytical levels and classifies evidence as method-matched thresholds, local or reference benchmarks, relative or temporal comparisons, mechanistic evidence and plant–soil response validation. The reviewed evidence shows that standardized measurements do not necessarily support transferable diagnostic criteria and that volcanic-soil and andic properties can affect both analytical behavior and the functional meaning of indicator values. Rather than assigning universal scores, indicators are integrated by soil function and interpreted in relation to soil mineralogy, crop requirements, management history, and disturbance or recovery trajectories. This approach helps distinguish intrinsic volcanic-soil properties from management-induced degradation, identify functionally relevant constraints and make diagnostic confidence explicit. The framework provides a practical basis for routine monitoring, targeted diagnosis, and the development of locally validated soil health reference systems in volcanic island agriculture.

## 1. Introduction

Soil health assessment has become a central component of sustainable land management, agronomic decision-making and environmental monitoring. The concept is commonly framed in functional terms, referring to the capacity of soil to sustain biological productivity; maintain environmental quality; and support plant, animal and human health [1,2]. In agricultural systems, soil health assessment is increasingly used to identify constraints to productivity, evaluate the effects of management practices, monitor degradation or recovery trajectories, and support more resilient agroecosystems [3,4,5].

Most soil health assessment frameworks rely on a combination of physical, chemical and biological indicators. These indicators provide measurable proxies for soil functions that cannot be assessed directly through a single variable, such as nutrient cycling, water regulation, structural stability, biological activity and plant-support capacity [3,5]. However, the diagnostic value of an indicator depends not only on the accuracy of the measurement, but also on the robustness of its interpretation. Some variables can be evaluated using relatively well-established agronomic or environmental thresholds, whereas others require comparison with local reference values, management gradients, paired control-impact designs or temporal trajectories [5,6].

This distinction is particularly relevant in volcanic island agroecosystems. Volcanic soils, especially Andosols and other soils with pronounced andic properties, often display distinctive mineralogical, chemical and physical properties, including short-range-order minerals, variable-charge surfaces, active Al and Fe phases, strong organo-mineral interactions, high phosphorus retention, low bulk density, high porosity and high water-holding capacity [7,8,9,10]. These properties strongly influence carbon stabilization, nutrient availability, aggregate formation, water regulation and biological functioning. Consequently, values that would be interpreted as optimal, deficient or degraded in other soil types may have a different functional meaning in volcanic soils.

In island agroecosystems, this intrinsic soil complexity is combined with limited agricultural land; high spatial heterogeneity; dependence on irrigation and fertilization; intensive production systems; salinity risk; and, in active volcanic regions, direct disturbance by ash deposition or ash incorporation [11,12,13,14]. Soil health assessment in these systems must therefore distinguish inherent volcanic soil properties from management-induced degradation, short-term disturbance effects and longer-term functional recovery. Applying generic interpretive criteria without considering these factors may lead to oversimplified or misleading diagnoses.

Despite the availability of numerous soil health indicators and well-established analytical methods, there is still a methodological gap in how these indicators are selected, combined and interpreted for volcanic island agroecosystems. General soil health frameworks do not always account for the specific reactivity of volcanic soils, the role of andic properties, the dependence of exchange processes on pH, the complexity of water availability in highly porous soils, or the context-dependent nature of biological and functional indicators.

This article is conceived as a critical methodological review rather than a systematic or scoping review. The literature was selected to support the development of an operational framework for soil health assessment in volcanic island agroecosystems, with emphasis on the methodological use and interpretation of indicators. To improve the transparency of the evidence base, targeted literature searches were conducted in the Web of Science Core Collection and Scopus, without a lower publication-year restriction and updated to 21 August 2026. Search combinations covered seven complementary thematic areas: soil health assessment frameworks and indicator interpretation; volcanic and andic soils in relation to soil health assessment; chemical and mineralogical method suitability; physical and hydrological method suitability; biological and functional indicators; fresh volcanic ash, disturbance and recovery; and agricultural contexts relevant to islands. The complete search strategy, including the database-specific search strings and document-type restrictions, is provided in Table S1. Searches were designed to identify methodologically and interpretively relevant evidence rather than to provide an exhaustive inventory of publications. Peer-reviewed articles and reviews were prioritized, while seminal methodological publications and authoritative standards, technical guidance and regulatory sources were consulted directly when required to define analytical procedures, operationally measured fractions or interpretation criteria.

Evidence was classified according to whether it supported method-matched diagnostic thresholds or crop-response relationships, crop-, method- or region-specific critical values, reference ranges or peer-group benchmarks, relative or temporal interpretation, methodological adaptations or limitations, mechanistic or contextual interpretation, plant–soil functional validation, or disturbance and recovery assessment. Publications were retained when they provided direct methodological or interpretive evidence relevant to indicator selection, analytical suitability, interpretation, functional validation or disturbance/recovery assessment; purely descriptive studies without a material contribution to these components were not used as primary evidence. Representative studies were selected to illustrate procedures for which volcanic-soil properties create a documented or mechanistically supported risk of analytical bias, sample-preparation bias or operational mismatch, as well.as the principal forms of interpretive evidence and their transferability and is not intended as an exhaustive compilation of published thresholds or reference values.

Because the literature considered spans different soil-classification systems and disturbance contexts, the terminology used in this article is deliberately differentiated. “Volcanic soils” is used as a broad, non-taxonomic term for soils whose properties are substantially influenced by volcanic parent materials or tephra. “Andosol” refers specifically to the Reference Soil Group defined by the World Reference Base for Soil Resources (WRB), whereas “soils with andic properties” refers to soils in which the corresponding diagnostic properties are expressed, without implying that the whole soil profile necessarily classifies as an Andosol [10]. The term “Andisol” is retained when it is the classification used in studies based on United States Department of Agriculture (USDA) Soil Taxonomy and is not treated as taxonomically interchangeable with WRB Andosol. Fresh volcanic ash and ash-affected soils are considered separately as disturbance contexts rather than as synonyms for pedogenically developed volcanic soils or soils with andic properties.

The aim of this review is to develop an integrated methodological framework for soil health assessment in volcanic island agroecosystems by linking soil functions, indicator selection, method suitability, analytical workflows and interpretation criteria. This review argues that robust assessment in these systems requires a stepwise and context-sensitive approach, in which analytical results are interpreted in relation to soil properties, crop requirements, management history and disturbance or recovery trajectories.

## 2. Context Dependence in Soil Health Assessment of Volcanic Island Agroecosystems

The interpretation of soil health indicators in volcanic island agroecosystems is conditioned by distinct but interacting levels of context. The first comprises properties associated with volcanic parent materials and, where expressed, andic properties; these controls are relevant to volcanic soils irrespective of whether they occur in island settings. The second comprises pressures associated with island agriculture, including limited land availability, irrigation dependence, intensive fertilization, salinity risk and highly managed production systems. Recent volcanic disturbance adds a third, temporally dynamic context through ash deposition, incorporation and post-eruption recovery. These dimensions do not necessarily require a completely different set of indicators, but they do require explicit contextualization of indicator selection and interpretation.

In volcanic soils, several standard indicators may reflect inherent soil-forming processes rather than recent changes in management. High organic carbon content, low bulk density, high porosity and high water-holding capacity, for example, are often associated with andic properties and strong organo-mineral interactions [7,8,9,10]. In many non-volcanic agricultural soils, these values may be interpreted as signs of favorable structure or improved management. In volcanic soils, however, they may partly represent intrinsic pedogenic features. Conversely, indicators such as method-defined soil-test phosphorus, cation exchange capacity or exchangeable cations may be strongly influenced by phosphate retention, variable-charge surfaces, pH-dependent exchange reactions and active Al and Fe phases [8,9]. Their interpretation therefore requires knowledge of the mineralogical and chemical reactivity of the soil, not only comparison with generic agronomic thresholds.

A second challenge concerns the interpretation of physical and hydrological indicators under agricultural use. Volcanic soils may retain large amounts of water [7,8], but high water retention does not automatically imply adequate water availability for crops. Soil-water functioning also depends on pore-size distribution, drainage, hydraulic conductivity, aeration and the ability of the root zone to supply water between irrigation events. This is particularly relevant in irrigated perennial systems, where crop performance is affected by the interaction among soil texture, root-zone structure, rooting depth, irrigation scheduling, drainage and salinity risk. For this reason, indicators such as water retention, available water capacity, infiltration, drainage and air-filled porosity should be interpreted together rather than as independent physical descriptors. Studies conducted in cultivated volcanic soils from Tenerife have shown that water retention, hydraulic conductivity, salinity, sodicity and residual andic properties interact in determining soil physical functioning, whereas infiltration across volcanic soils of different developmental stages is controlled by different combinations of structural development, aggregate stability and particle-size distribution [15,16]. Similarly, work on an irrigated Chilean Andosol demonstrated that high plant-available water can support pasture production and reduce irrigation requirements, but only when water-storage characteristics and soil-moisture measurements are calibrated for the specific soil and rooting system [17].

A third dimension is the agricultural context of islands. Limited land availability, high spatial heterogeneity and dependence on irrigation, fertilization, and external or locally recycled inputs can intensify soil constraints. Electrical conductivity, exchangeable sodium, soil-test phosphorus or organic matter content may indicate different processes depending on management history. The same value may reflect natural variability, fertilization legacy, restricted drainage, organic amendment use, irrigation-water quality, volcanic ash incorporation or combinations of these factors. In this context, soil health assessment requires explicit consideration of the production system and of the management question being addressed.

Volcanic disturbance adds a temporal dimension to soil health interpretation. Ash deposition may generate short-term constraints by altering surface conditions, infiltration, aeration, pH, salinity, nutrient availability and plant establishment. However, volcanic ash is also a parent material that can contribute to soil development and fertility after weathering, leaching and incorporation into the soil matrix [8,13,14]. This dual role means that ash-affected soils should not be assessed only through static comparisons with generic reference values. Interpretation should distinguish among acute disturbance, management-induced degradation, functional adaptation and post-disturbance recovery. Medium-term observations after ash deposition also show that recovery trajectories are indicator- and land-use-dependent: surface litter, aggregate stability and infiltration may recover more rapidly than soil organic carbon (SOC) accumulation in newly deposited ash, particularly under forest or agroforestry vegetation [18].

Together, these contextual dimensions affect both the expected values of soil indicators and the evidential basis required to interpret them. The framework developed below therefore separates contextual characterization from the subsequent selection of functionally relevant indicators and interpretation criteria.

## 3. A Function-Based and Interpretation-Oriented Framework for Indicator Selection

Building on the contextual conditions defined in Section 2, the proposed framework organizes indicator selection along two complementary axes: functional relevance and interpretation basis [3,5,6]. The first identifies the soil function or diagnostic role that a measurement is intended to inform; the second specifies the type of evidence available to interpret the resulting value.

The functional axis prevents indicators from being treated as isolated analytical variables. Measurements are selected to inform nutrient cycling and chemical fertility, salinity and ion balance, water regulation, structural stability and biological functioning, while volcanic-soil and andic properties may provide mechanistic or contextual information, and plant or temporal responses may provide functional validation. Previous indicator-selection work in temperate humid volcanic soils of southern Chile illustrates this approach. From a broader set of chemical and physical properties, Valle and Carrasco selected a reduced group combining dynamic indicators with volcanic-soil-specific controls, including bulk density, plant-available water and air-related pore functions, SOC, extractable Al, pH, Olsen-P and base status [19].

The interpretive axis requires the intended interpretation basis to be specified before indicators are integrated. Depending on the available evidence and assessment objective, measurements may support method-matched diagnostic thresholds, reference ranges, relative comparisons, mechanistic or contextual interpretation, plant–soil validation, or temporal trajectories. These categories are summarized in Table 1 and examined in detail in Section 5. They are not mutually exclusive, because the same measurement may serve different interpretive roles depending on the diagnostic question.

**Figure 1:**
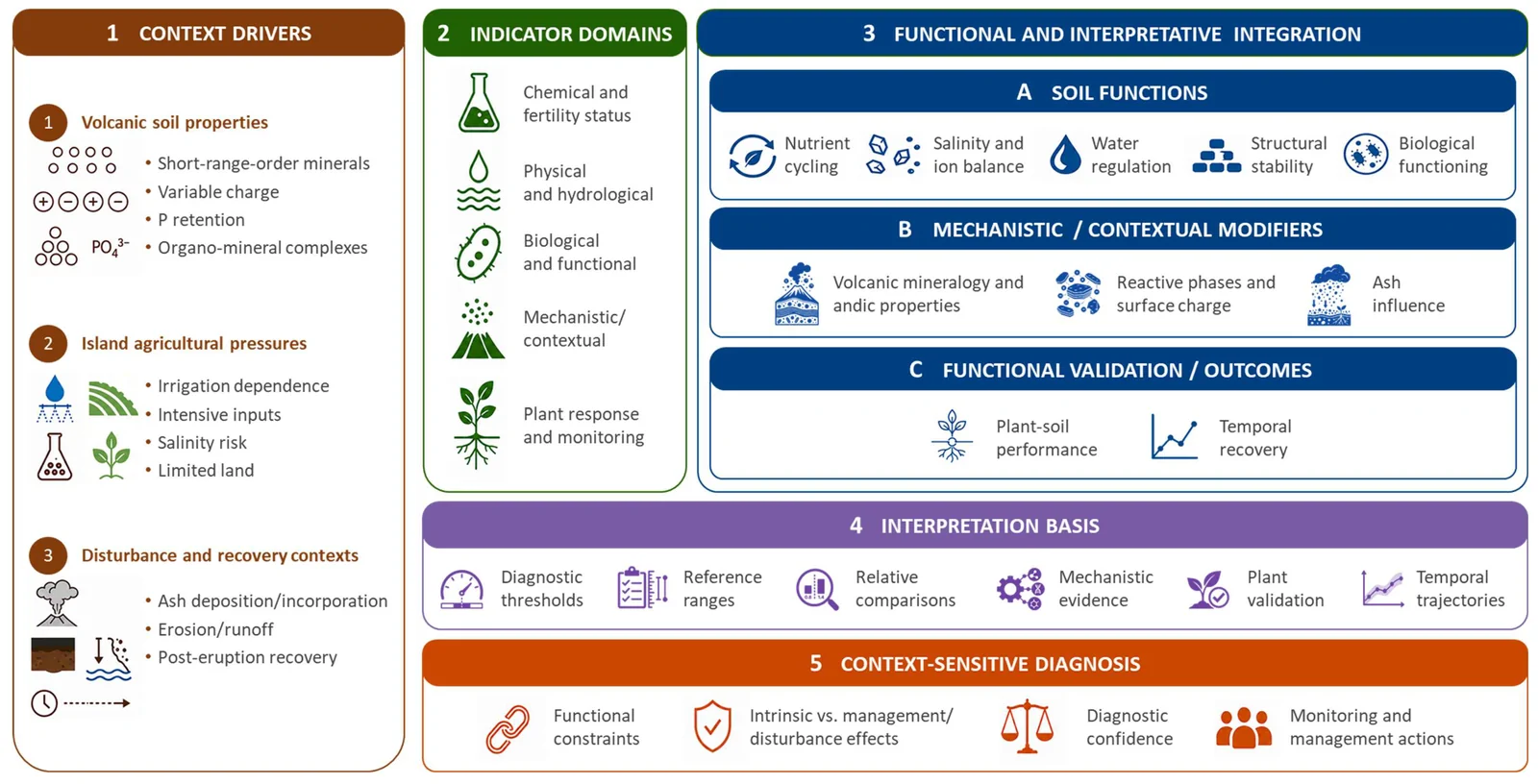
Conceptual framework for context-sensitive soil health assessment in volcanic island agroecosystems. Context drivers guide the selection of indicator domains. Measurements are integrated according to the soil functions under assessment, while volcanic-soil and andic properties provide mechanistic or contextual modifiers and plant or temporal responses provide functional validation. Interpretation is based on method- and context-appropriate evidence before a context-sensitive diagnosis and associated confidence level are established.

Figure 1 summarizes how context drivers, indicator domains, functional roles and interpretation categories are combined before a context-sensitive diagnosis is produced. The analytical depth required to generate those measurements is considered separately in Section 4.

## 4. Indicator Domains and Analytical Levels for Context-Sensitive Assessment

The framework proposed above requires translating soil functions and interpretation categories into practical indicator domains. In volcanic island agroecosystems, this translation should not be based on the idea that all possible indicators must be measured in every assessment. Instead, indicator selection should be proportional to the assessment objective, the level of diagnostic uncertainty and the resources available. A routine monitoring program, a fertility or salinity diagnosis, an evaluation of volcanic ash-affected soils and an advanced mechanistic study do not require the same analytical depth. Analytical adaptations driven by volcanic mineralogy or andic properties are relevant to volcanic soils in both island and continental settings, whereas island-specific conditions primarily modify assessment priorities, sampling context and the interpretation of management, irrigation, salinity and disturbance effects.

For this reason, indicators can be organized into complementary analytical levels: a core routine screening dataset, a baseline soil health dataset, an expanded functional dataset, a mechanistic/contextual dataset, an integrated plant–soil response dataset and a disturbance and temporal monitoring dataset. These levels are not mutually exclusive; they define a stepwise strategy in which core screening can be expanded to a broader baseline assessment and more specialized measurements are added only when required by the diagnostic question. The proposed indicator domains and analytical levels are summarized in Table 2, which is intended as a methodological guide rather than a fixed checklist. Whereas Table 1 classifies indicators according to how their results should be interpreted, Table 2 organizes the analytical effort required to generate those results according to the assessment objective and diagnostic uncertainty.

### 4.1. Core Screening and Baseline Soil Health Indicators

Core routine screening is intended to identify major first-line agronomic constraints using a small set of broadly accessible measurements rather than to provide a complete assessment of soil health. Depending on the assessment objective, this level may include pH; electrical conductivity (EC); soil organic carbon (SOC) or a locally calibrated loss-on-ignition (LOI) estimate; method-defined soil-test P; and selected nutrient, exchangeable-cation or soluble-ion measurements required to address the principal fertility or salinity question.

A baseline soil health assessment extends this screening across chemical, physical and biological domains. Additional variables may include total nitrogen (TN), potential and/or effective cation exchange capacity (CEC), exchangeable cations, particle-size distribution determined using a validated dispersion procedure, coarse-fragment content, bulk density, aggregate stability, water-holding capacity (WHC) and/or available water capacity (AWC), where appropriate and explicitly defined, and a basic biological indicator such as basal respiration or substrate-induced respiration (SIR) [3,5,7,8,19].

These indicators are intended to identify major constraints and establish a baseline rather than provide stand-alone evidence of improvement or degradation. Dry combustion directly determines total carbon; it represents SOC only where inorganic carbon is absent or negligible. Where carbonates or other inorganic C sources are present, SOC should be determined by correcting total carbon for independently quantified inorganic carbon or by using a validated carbonate-removal pretreatment before combustion. The analytical state or fraction measured should be identified explicitly: soil-test P should be identified by the extraction method used and interpreted only against method-compatible criteria; WHC should be identified as an operational water-retention measurement defined by the specific wetting, drainage and measurement procedure used, whereas AWC should be calculated from water contents at explicitly stated upper and lower matric-potential limits; LOI should not be treated as equivalent to SOC without local calibration; CEC determined with buffered ammonium acetate (NH_4_OAc) at pH 7 represents potential rather than field-state exchange capacity; and particle-size results should not be assumed to represent primary soil texture unless adequate dispersion has been demonstrated. Procedures requiring adaptation or explicit qualification in volcanic soils are examined in Section 4.6 and Table 3.

### 4.2. Expanded Functional Indicators

Expanded functional indicators are required when the objective is to evaluate soil processes rather than only describe the baseline condition. This level is particularly relevant for comparing management practices, detecting early degradation, evaluating biological recovery or investigating soil or crop responses that are not explained by routine chemical indicators. Relevant measurements may include water-retention parameters, plant-available water estimates, infiltration, saturated or unsaturated hydraulic conductivity, water repellency, drainage or aeration indicators, respiration-derived indices, metabolic quotients, community-level physiological profiles (CLPP) [20,21,22,23], enzyme activities [24,25], arbuscular mycorrhizal fungi (AMF) indicators [26,27,28] and glomalin-related soil proteins [29,30,31,32].

Studies conducted specifically in volcanic soils demonstrate the value of combining these indicators. In Chilean Andisols, land use and management have been evaluated through bulk density, pore functions, plant-available water, air conductivity, aggregate behavior, SOC and reactive Al [19,33]. In Tenerife, infiltration and physical functioning have been related to structural development, aggregate stability, particle-size distribution and residual andic properties [15,16], whereas irrigation studies in a Chilean Andosol have linked water retention and soil-moisture dynamics with pasture response and mechanical resistance [17].

Expanded functional indicators should therefore be selected according to the process under investigation and measured under standardized, method-compatible conditions. Their interpretation basis—reference range, relative comparison or temporal trajectory—is examined in Section 5.

### 4.3. Mechanistic Indicators of Volcanic-Soil and Andic Properties

In many volcanic soils, conventional indicators cannot be fully interpreted without information on the mineralogical, chemical and surface-reactivity controls associated with volcanic parent materials and, where expressed, andic properties. These mechanistic measurements are used mainly to explain why a soil shows particular values of phosphorus availability, carbon stabilization, cation exchange capacity, aggregation, water retention or biological response [7,8,9,10]. Relevant measurands include phosphorus retention; NaF reactivity; operationally extractable Al, Fe and Si fractions; mineralogical composition; specific surface area; surface-charge behavior; and microstructural or local elemental characteristics. These properties should be distinguished from the analytical procedures or techniques used to determine them, such as selective extraction followed by elemental determination, X-ray diffraction, gas adsorption, electrokinetic measurements or microscopy coupled with elemental analysis. Selective Al, Fe and Si extractions should be interpreted as operationally defined fractions rather than as direct quantitative measurements of unique mineralogical or chemical species. Oxalate-extractable pools can provide evidence of poorly crystalline or short-range-order reactive phases, whereas pyrophosphate-extractable fractions can support interpretation of organo-metal associations, but neither extraction is uniquely specific to a single mineral or chemical pool.

X-ray diffraction (XRD) is useful for identifying crystalline mineral phases, but short-range-order materials that are central to many soils with andic properties may be weakly represented or poorly resolved by conventional XRD. Scanning electron microscopy coupled with energy-dispersive X-ray spectroscopy (SEM–EDS) provides complementary information on microstructure and local elemental associations, but these observations should not be interpreted as representative bulk quantitative composition or as independent quantification of allophane, imogolite or related reactive phases. XRD and SEM–EDS should therefore be used as complementary mechanistic tools and interpreted together with selective dissolution and other mineralogical or geochemical evidence. Where representative bulk elemental composition is required to resolve a mechanistic question, complementary approaches such as X-ray fluorescence or inductively coupled plasma-based elemental analysis following an explicitly defined digestion may be incorporated; concentrations obtained after digestion should be identified as total or pseudo-total according to the extent of sample decomposition.

These measurements are not required for every routine assessment. They should be added when routine indicators are ambiguous, particularly when phosphorus availability, organic carbon stabilization, cation exchange processes, water retention or biological responses cannot be explained without information on volcanic or andic reactivity.

### 4.4. Integrated Plant–Soil Response Indicators

Soil health assessment becomes more robust when soil indicators are connected with plant performance. Integrated plant–soil response indicators include crop biomass, root traits, foliar nutrient status, leaf chlorophyll status assessed by direct pigment analysis or Soil Plant Analysis Development (SPAD) readings, gas exchange, stomatal conductance, water potential, visual symptoms and mycorrhizal response. These variables help determine whether changes in soil properties are functionally relevant for crop growth or physiological performance [5,6]. This type of integrated design has recently been applied to volcanic ash–soil mixing, combining plant growth and architecture, soil physicochemical indicators, respiration-based microbial indices and leaf ionomics to detect early functional responses during crop establishment [34].

Plant-response indicators are particularly useful when soil thresholds are uncertain or when multiple constraints interact. In volcanic island agroecosystems, plant performance may reflect the combined effects of salinity, water limitation, phosphorus retention, ash incorporation, particle-size distribution, rooting conditions and biological functioning. In these situations, plant-based measurements help validate whether an apparent soil constraint is expressed as an agronomic or physiological response.

### 4.5. Fresh-Ash Characterization and Temporal Monitoring Indicators

Where freshly deposited volcanic ash is part of the assessment, it should initially be characterized as a distinct material rather than treated as equivalent to the underlying soil or to a developed volcanic soil. In addition to pH and electrical conductivity, fresh-ash assessment may include readily water-leachable major ions and potentially phytotoxic soluble species where relevant to the eruption and agricultural receptor [13,14]. Ash-leachate results are operational measurements and should therefore be accompanied by the ash-to-water ratio; extraction or contact time; extraction sequence, where applicable; filtration procedure; and analytical method used, because these conditions can substantially affect the measured concentrations [14].

Temporal monitoring indicators are required when soil health assessment aims to evaluate degradation, adaptation or recovery trajectories rather than a single static condition. This level is particularly important in volcanic island agroecosystems affected by ash deposition, ash incorporation, erosion, salinity episodes, irrigation changes, restoration practices or major shifts in management [12,13,14,18]. In these contexts, one sampling date may be insufficient to distinguish acute disturbance from transient adjustment, functional recovery or longer-term soil development. Post-eruption designs should therefore distinguish, where these materials remain identifiable, the fresh ash layer, the underlying pre-existing soil and mixed ash–soil horizons, and should follow their evolution as leaching, weathering and incorporation proceed.

Temporal monitoring should prioritize a consistent subset of indicators that is sensitive to the expected process and reproducible across dates. Depending on the material and stage of recovery, repeated measurements may include pH, EC, soluble ions, method-defined soil-test nutrients, SOC, aggregate stability, water retention, infiltration, microbial activity and plant response. The recovery study conducted after the 2014 Mt. Kelud eruption illustrates this approach: SOC, aggregate stability, porosity and infiltration followed different trajectories over six years and differed among forests, agroforestry systems and annual crops [18]. Interpretation should therefore focus on the direction, rate and persistence of change rather than on isolated values.

### 4.6. Analytical Procedures Requiring Adaptation in Volcanic Soils

The selection of an appropriate indicator does not guarantee that a conventional analytical procedure will provide an unbiased measurement in volcanic soils, particularly those expressing andic properties. Short-range-order minerals, organo-Al complexes, variable-charge surfaces, highly stable organo-mineral aggregates and hydrated pore structures can interfere with sample dispersion, exchange measurements, drying and thermal pretreatments. Consequently, methods standardized for soils dominated by crystalline layer silicates may measure an operationally different property when applied without modification to volcanic soils.

Three distinct methodological problems should be differentiated. Analytical incompatibility occurs when the procedure does not adequately isolate or measure the intended property, as may occur when routine dispersion fails to release primary particle-size fractions. Operational mismatch occurs when a valid method measures a different state or fraction from the one assumed in the interpretation, such as potential CEC at pH 7 being reported as effective CEC. Sample-preparation bias occurs when drying, heating, grinding or repacking modifies the property before it is measured. These problems differ from interpretive non-transferability, in which the analytical result is valid but the associated threshold or scoring function is not applicable to volcanic soils.

Representative procedures requiring adaptation or explicit qualification are summarized in Table 3. The table is not intended as an exhaustive catalogue of soil methods. It identifies cases for which volcanic-soil properties create a documented risk of analytical bias or of misidentifying the measured property.

#### 4.6.1. Particle-Size Distribution and Soil Texture

Particle-size analysis is particularly problematic in volcanic soils because the conventional sedimentation approach assumes that primary mineral particles have been completely dispersed before their settling behavior is measured. In allophanic soils, aggregates formed by short-range-order minerals, reactive Al and Fe phases and organic matter may remain stable after routine treatment with hydrogen peroxide, chemical dispersants and mechanical shaking [35,36,37,38]. These resistant aggregates may be recorded as apparent sand or silt, resulting in underestimation of the clay-sized fraction.

In oxidic and volcanic ash soils from Hawai’i, increasing ultrasonic energy substantially increased measured clay contents, whereas increasing the concentration of the conventional sodium hexametaphosphate dispersant produced only limited improvement [35]. The energy required for dispersion depended on soil organic carbon, oxide content and surface charge. In an allophanic Andisol, maximum dispersion was obtained only after Na saturation followed by sonication at substantially higher energy than normally required for non-volcanic soils [36]. These results demonstrate that routine Bouyoucos or pipette procedures cannot be assumed to produce primary particle-size distributions unless adequate dispersion has been verified.

Differences are also observed in non-allophanic volcanic soils. Comparison of hydrometer, pipette and laser-diffraction methods in Patagonian volcanic soils showed method-dependent estimates of sand, silt and clay. The hydrometer method tended to overestimate sand and underestimate silt relative to the pipette method, partly because incompletely dispersed fine particles occurred as sand-sized pseudo-aggregates [37]. Ignition at 430 °C to remove organic matter before particle-size analysis was not recommended because heating could alter mineral particles and did not provide a reliable correction [37].

Laser diffraction should not be considered an automatically valid alternative. Its results still depend on dispersion, particle shape, refractive-index assumptions and the conversion of optical diameter into equivalent particle-size classes. Comparisons with sedimentation methods have shown particularly poor agreement for the clay fraction in volcanic materials [35,37]. The complete analytical sequence—sample pretreatment, dispersion and particle-size measurement—must therefore be validated.

Where primary particle-size distribution is required, dispersion should be evaluated experimentally by applying increasing dispersive treatments until the released fine fraction approaches a plateau, while ensuring that primary particles are not damaged. Appropriate treatments may include Na saturation or alkaline dispersants combined with controlled sonication, depending on soil mineralogy [35,36]. The sand fraction should preferably be determined gravimetrically by sieving. When complete dispersion cannot be demonstrated, results should be described as an operational particle-size distribution rather than assumed to represent true soil texture.

#### 4.6.2. Potential and Effective Cation Exchange Capacity

Cation exchange capacity in volcanic soils is strongly dependent on the pH and ionic strength imposed during measurement. The widely used 1 M ammonium acetate method buffered at pH 7 generates additional negative charge on allophane, imogolite, Al–humus complexes and soil organic matter. It therefore measures a potential CEC under the imposed analytical conditions rather than the charge expressed at field soil pH.

Studies of Andisols and soils with andic properties from the Canary Islands showed that the relative difference between potential CEC determined with NH_4_OAc at pH 7 and actual CEC determined under conditions closer to field pH exceeded 70% in some soils with low contents of layer silicates [39]. Potential CEC was positively associated with oxalate-extractable Al, whereas actual CEC showed a different relationship, illustrating that the two measurements describe different aspects of the variable-charge system. Comparable differences among ammonium acetate and compulsive-exchange methods have been reported for Andisols from the Azores [40].

The NH_4_OAc method should not therefore be discarded, but its result must be identified explicitly as potential CEC at pH 7. When the objective is to characterize nutrient retention or exchange status under current field conditions, it should be complemented by an estimate of effective or actual CEC obtained using an unbuffered procedure at or near soil pH. Suitable alternatives include the compulsive-exchange or silver-thiourea methods, or an effective CEC calculated from exchangeable bases and acidity when the extraction procedures are clearly specified [39,40]. Potential and effective CEC should not be used interchangeably or interpreted against the same reference values.

#### 4.6.3. Drying, Rewetting and Alteration of Field-State Properties

Drying is not a neutral sample-preservation step in many volcanic soils. Dehydration of short-range-order materials, strengthening of organo-mineral associations and rearrangement of highly porous aggregates can produce changes that are only partly reversed after rewetting. These effects can influence particle dispersion, water retention, aggregate behavior and operationally extractable chemical fractions.

In Andisols from Mount Kawi, air-drying and oven-drying at 40 °C altered measured water content, water-dispersible clay, organic C and the operationally defined available-P fraction reported by the authors [41]. Rewetting did not restore water content or dispersed-clay values to their initial field-moist condition, indicating partially irreversible alteration. The reported P fraction also changed after drying, showing that sample history can affect operational nutrient fractions in soils containing reactive amorphous materials.

Air-drying may also alter the distribution of soil organic matter among density fractions. In organic horizons containing volcanic ash and reactive Al and Fe phases, air-drying changed the recovery of mass, organic C and total N among density fractions relative to field-moist samples [42]. Freeze-drying produced smaller changes in two of the three soils examined, although it was not completely neutral in all samples. Earlier studies similarly showed that drying can increase the resistance of allophanic aggregates to ordinary dispersion treatments [38].

**Table 3:** Selected analytical procedures requiring adaptation or explicit qualification in volcanic soils and soils with andic properties.

Target Property	Key Analytical Risk in Volcanic Soils	Recommended Practice/Reporting	Sources
Particle-size distribution and soil texture	Resistant short-range-order and organo-mineral aggregates may prevent complete dispersion; charge, particle shape and heating can bias results	Verify dispersion to a plateau; consider Na saturation/alkaline dispersants plus controlled sonication; determine sand gravimetrically; avoid ignition; validate laser diffraction; report an operational particle-size distribution if complete dispersion is not demonstrated	[35,36,37,38]
Cation exchange capacity	Buffered NH_4_OAc at pH 7 increases negative charge and measures potential rather than field-state CEC	Report explicitly as potential CEC at pH 7; where field exchange status is required, complement with an unbuffered estimate of effective/actual CEC at or near soil pH	[39,40]
Water retention, hydraulic and structural properties	Drying, rewetting and repacking may irreversibly alter pore structure and aggregation	Prefer field-moist samples and minimally disturbed cores for in situ functioning; report drying/rewetting history; use dried or repacked material only for clearly defined operational measurements	[38,41]
Method-defined extractable P, water-dispersible clay and SOM fractions	Drying can modify reactive surfaces, aggregation and operationally extractable or fractionated pools	Match pretreatment to the calibration or study objective; use field-moist material for field-state fractions; validate freeze-drying where used; report storage and drying history	[38,41,42]
LOI-based SOM/SOC estimation	Structural water and mineral dehydroxylation contribute to ignition loss, making universal conversion factors unreliable	Standardize ignition conditions and calibrate LOI against SOC determined from dry-combustion carbon analysis with inorganic C appropriately accounted for; do not apply universal LOI-to-SOM or LOI-to-SOC conversion factors	[7,8,9,43]

The table summarizes selected procedures for which volcanic-soil properties may introduce analytical bias or operational mismatch. Operational measurements remain valid when the measured property, sample pretreatment and analytical conditions are explicitly reported and interpretation criteria are matched to the same procedure. Standardized agronomic tests using air-dried soil remain operationally valid when the corresponding calibration was developed using the same pretreatment. Abbreviations: CEC, cation exchange capacity; LOI, loss on ignition; NH_4_OAc, ammonium acetate; SOC, soil organic carbon; SOM, soil organic matter.

Field-moist samples and minimally disturbed cores should therefore be used when the objective is to represent in situ water retention, hydraulic functioning, pore structure, water-dispersible particles or field-state organo-mineral associations. Drying history, storage temperature, duration and rewetting procedure should be reported. Samples that have been air-dried or oven-dried and subsequently repacked remain useful for standardized operational measurements, but the resulting values should not be interpreted as direct representations of undisturbed field behavior.

For agronomic soil tests conventionally calibrated using air-dried soil, drying does not automatically invalidate the result. However, the same sample pretreatment used to develop the calibration must be followed, and values derived from air-dried and field-moist samples should not be compared without method-specific validation.

#### 4.6.4. Loss on Ignition as an Estimate of Soil Organic Matter or Carbon

Loss on ignition is frequently used as a low-cost estimate of soil organic matter (SOM), but mass loss during heating does not arise exclusively from organic matter combustion. It may also include dehydration and dehydroxylation of mineral phases, carbonate decomposition and other temperature-dependent reactions. Ignition temperature, duration, furnace conditions, sample mass and mineral composition can therefore influence the result [43].

This limitation is potentially important in volcanic soils because allophane, imogolite, ferrihydrite and related short-range-order phases contain substantial structural and adsorbed water [7,8,9]. However, direct evaluations of universal LOI-to-SOC conversion factors across representative volcanic-soil mineralogies remain limited. LOI should consequently be regarded as an operational mass-loss measurement rather than a direct determination of SOC.

Dry combustion should be preferred as the basis for quantitative SOC determination or C-stock assessment, but the measured carbon fraction must be defined explicitly. Dry combustion directly determines total carbon; where inorganic carbon is absent or negligible, total carbon can be used as SOC, whereas carbonate-containing soils require either independent quantification of inorganic carbon and subtraction from total carbon or a validated carbonate-removal pretreatment before combustion [44]. If LOI is used for routine monitoring, ignition conditions should be strictly standardized and a soil-specific calibration against SOC determined using this explicitly defined combustion-based procedure across the relevant range of mineralogy and organic matter content. Universal conversion factors between LOI, soil organic matter and SOC should not be applied without validation.

Overall, the use of adapted procedures does not imply abandoning methodological standardization. Rather, standardization should begin by defining the intended property or operationally defined fraction, confirming that sample preparation does not alter it, and verifying that the analytical conditions are compatible with volcanic-soil mineralogy. Where a method measures an operational or potential property, that property should be named explicitly and interpreted only using criteria developed for the same procedure.

## 5. Evidence Basis for Diagnostic Interpretation

The distinction between analytical level and interpretation category is essential because the availability of a standardized method does not necessarily imply the existence of a validated diagnostic criterion. Published interpretation approaches range from relatively direct thresholds linked to defined agronomic constraints to statistical benchmarks, local comparisons and temporal trajectories. Their diagnostic strength depends on the relationship between the measured indicator and the functional outcome of interest, but also on analytical method, units, sampling depth, crop, soil type, climate and management context.

Transferability therefore needs to be considered at two complementary levels. Volcanic-soil and andic properties may alter indicator values, analytical behavior or mechanistic interpretation irrespective of geographic setting, whereas island-specific crop, irrigation, salinity, management and disturbance contexts determine whether otherwise valid criteria are locally applicable. The following sections distinguish the principal forms of interpretive evidence.

### 5.1. Indicators Supported by Direct Diagnostic Thresholds

Direct diagnostic thresholds are most defensible when an indicator is linked to a clearly defined agronomic or environmental constraint and when the measurement method used is the same as that for which the threshold was established. Salinity and sodicity provide the clearest examples. The classical diagnosis of saline and sodic soils was developed using the electrical conductivity of the saturated-paste extract (ECe), exchangeable sodium percentage (ESP) and sodium adsorption ratio (SAR) [45]. Historically, an ECe of 4 dS m^−1^ was used as a general boundary for saline soils and an ESP of 15% as a boundary for sodicity. These values remain useful operational reference points, but they should not be interpreted as universal biological discontinuities. Crop effects may occur below or above these values depending on crop tolerance, growth stage, rooting conditions, irrigation regime, salt composition and drainage.

A critical methodological requirement is that thresholds should not be transferred among analytical procedures without calibration. An ECe threshold cannot be applied directly to EC measured in a 1:2, 1:2.5 or 1:5 soil–water suspension because dilution, soil texture, soluble-ion composition and gypsum or carbonate content alter the relationship among methods. The extraction procedure, soil-to-solution ratio, temperature correction and units should therefore accompany every salinity diagnosis. Similar considerations apply to SAR calculated from irrigation water, soil solution or saturation extract, and to ESP derived using different procedures for cation exchange capacity and exchangeable Na.

Soil pH can also support direct diagnosis when values indicate a plausible risk of severe acidity, alkalinity, Al or Mn toxicity, micronutrient deficiency, or impaired nutrient availability. However, the boundaries of the favorable range vary among crops and should not be interpreted independently of the pH method, soil mineralogy and exchange complex. In variable-charge volcanic soils, pH also regulates cation exchange capacity, anion retention, Al activity, phosphorus sorption and organo-mineral interactions. Consequently, pH may act simultaneously as a direct constraint indicator and as a mechanistic variable explaining the behavior of other indicators.

Thresholds for potentially toxic elements, soluble ions or nutrient deficiencies can likewise support direct diagnosis when they are based on an established regulatory criterion or a calibrated relationship with plant or environmental response. Nevertheless, total, pseudo-total, operationally extractable and soil-solution concentrations represent different analytical fractions or exposure domains and should not be evaluated against the same threshold. A concentration obtained after aqua-regia or another incomplete digestion should be identified as pseudo-total rather than assumed to represent the total elemental concentration, particularly in volcanic soils containing resistant silicate phases. Total concentrations require a procedure intended to achieve complete sample decomposition, such as complete digestion or fusion. Regulatory criteria and methodological guidance should be distinguished explicitly. Directive 86/278/EEC establishes limit values for specified trace elements in the context of sewage-sludge application to agricultural soils [46], whereas International Organization for Standardization (ISO) 22190 provides guidance on the use of soil extracts for assessing trace-element bioavailability and does not establish regulatory threshold values [47]. For potentially toxic elements in particular, pH, organic matter, mineralogy, redox conditions and competing ions may strongly modify mobility and plant availability.

Direct-threshold interpretation should therefore be restricted to cases in which the method and measured fraction match those used to derive the criterion, the threshold is linked to a defined functional outcome, and the relevant crop or environmental receptor and local soil context are represented. Otherwise, the numerical value should be treated as a provisional benchmark rather than a diagnostic boundary. Table 4 summarizes representative criteria and their transferability.

### 5.2. Crop-, Method- and Region-Specific Critical Values

Many agronomic indicators are interpreted through critical values rather than universal soil health thresholds. A critical soil-test value normally represents the concentration above which the probability or magnitude of crop response to an additional nutrient input becomes small. It is therefore an outcome-based criterion, but its validity is restricted to the soil-test method, crop, response variable, sampling depth, region and statistical model used to derive it. Accordingly, soil-test P values should be identified by the extraction method used (e.g., Olsen-P, Bray-P or Mehlich-extractable P), because these procedures quantify different operational fractions and their interpretive criteria are not interchangeable.

Phosphorus soil testing illustrates both the value and the limitations of this approach. In volcanic soils, P availability is strongly affected by short-range-order minerals, active Al and Fe phases, organo-mineral complexes and pH-dependent sorption [7,8,9,10]. Generic P categories derived from non-volcanic soils may therefore be poorly transferable. Nevertheless, crop-specific calibration is possible. Sandaña et al. [49] evaluated 41 P-fertilization experiments conducted between 1977 and 2015 in volcanic soils of southern Chile and estimated a critical Olsen-extractable P value of 33 mg P kg^−1^ for potato using a boundary-function approach. Importantly, the same dataset produced critical values of 11 and 41 mg P kg^−1^ when alternative response models were used. The study therefore demonstrates not only that useful critical values can be developed for crops grown on volcanic soils, but also that the selected statistical model and definition of adequate yield materially affect the resulting criterion. This example should not be interpreted as establishing 33 mg Olsen-P kg^−1^ as a general optimum for volcanic soils. The value was developed for potato, the Olsen extraction, a 0–20 cm sampling depth, volcanic soils of southern Chile, and the climatic and management conditions represented by those experiments. Its transfer to other crops or volcanic island regions would require validation. Differences in phosphate retention, rooting depth, irrigation, cultivar, yield target and soil sampling could modify the response relationship.

Comparable restrictions apply to critical values for exchangeable K, Ca and Mg; extractable micronutrients; mineral N; and plant-tissue concentrations. Soil-test interpretation is inseparable from the extractant used. Values generated with Olsen, Bray, Mehlich, ammonium acetate, diethylenetriaminepentaacetic acid (DTPA) or other operational procedures should only be compared with calibrations developed using the same or demonstrably equivalent method. The soil mass or volume basis, extraction ratio, shaking time and analytical determination may also influence results.

Crop salt-tolerance values represent another form of crop-specific critical criterion. The threshold–slope model developed by Maas and Hoffman [48] defines a crop-specific ECe below which no measurable yield reduction is expected and a subsequent percentage yield decline per unit increase in salinity. These values are more informative for crop diagnosis than a single general salinity boundary, but they remain conditional on crop species and cultivar, growth stage, salt composition, climate, soil-water regime and the way root-zone salinity was estimated. They should therefore be interpreted as agronomic response functions rather than intrinsic soil health classes.

Crop-, method- and region-specific critical values are among the most useful forms of evidence for decision-making because they connect a soil measurement with a measurable outcome. However, they diagnose a particular limitation or fertilizer response and should not be assumed to represent overall soil health. Their development for volcanic island crops would require coordinated calibration experiments linking standardized soil tests with yield, nutrient uptake, physiological response, input-use efficiency and environmental losses.

### 5.3. Reference Ranges and Peer-Group Benchmarks

For many dynamic soil properties, a single critical value is neither biologically realistic nor statistically defensible. Soil organic carbon, aggregate stability, active carbon, microbial respiration and available water capacity are strongly influenced by inherent soil-forming factors, as well as management. Their interpretation is therefore more appropriately based on reference distributions constructed for comparable soils.

The Comprehensive Assessment of Soil Health developed at Cornell converts measured values into scores using distributions from its calibration database [54]. Separate scoring functions are used for several physical and biological indicators according to broad textural groups, and indicator values are expressed relative to the distribution observed in the relevant comparison population. This approach recognizes that the expected value of soil organic matter, aggregate stability or available water capacity differs among coarse-, medium- and fine-textured soils. However, the resulting score is a statistical position within a calibration population rather than a universal functional threshold. The Cornell functions were initially developed largely from Northeastern United States’ soils and subsequently expanded, but they still require evaluation before being applied to markedly different climatic, mineralogical or land-use contexts.

The Soil Health Assessment Protocol and Evaluation, version 1.0 (SHAPE v1.0), provides a more explicitly context-dependent implementation of this principle. SHAPE v1.0 uses climate–edaphic peer groups and conditional cumulative distributions to interpret SOC, two wet-aggregate-stability measurements, permanganate-oxidizable carbon, autoclaved-citrate-extractable protein and four-day microbial respiration [55]. Measured values are expressed as quantiles within the relevant peer group, and benchmark values can be assigned at selected percentiles. This approach also allows uncertainty to be estimated and the difference between a measured value and a chosen benchmark to be expressed as an opportunity gap.

Regional adaptation of soil health assessment has already begun in some volcanic regions. In southern Chile, a minimum set of indicators was selected directly from volcanic soils representing different soil types, land uses and sampling dates [19]. In Hawai’i, Crow et al. evaluated 46 physical, chemical and biological properties across several islands, land uses and soil orders, including Andisols, and identified a reduced set of 11 indicators. Their results showed that mineralogy, current land use and the legacy of intensive plantation agriculture must be incorporated into the expected range and interpretation of indicator values [56].

Maaz et al. subsequently developed a structural-equation-model scoring approach that adjusts soil health scores for inherent soil properties and intensive land-use history, thereby reducing the risk that mineralogical or historical differences are attributed incorrectly to current management [57]. In Hawai’i Island Andisols, Beckstrom et al. applied this regional framework to SOM size and density fractions and dynamic soil properties across moisture regimes and land uses [58]. In southern Chile, comparisons between native forest and agricultural uses similarly showed that SOC loss and the response of bulk density, porosity, air capacity, plant-available water and pH differed among volcanic soil groups [59]. These studies demonstrate that volcanic-soil-specific benchmarking is feasible, but also that existing models remain regionally bounded.

Peer-group approaches are therefore preferable to universal scoring curves, but the comparison population must represent the relevant volcanic-soil context. Texture alone is insufficient because soils with similar particle-size distributions may differ substantially in short-range-order minerals, organo-Al complexes, specific surface area, aggregation and pore architecture. Reference groups may need to be stratified by soil group, degree of andic expression, parent material or ash influence, moisture regime, sampling depth, land use, crop system and management history.

SOC provides a clear example. A high SOC content may indicate effective organic inputs and C stabilization, but it may also be an intrinsic characteristic of an Andosol produced by Al–humus complexation and strong mineral protection [7,8,9,10]. A percentile developed from non-volcanic soils could consequently classify a volcanic soil as exceptionally favorable without showing that its current management is improving soil function. SOC should instead be evaluated relative to soils with comparable mineralogical and climatic potential, and preferably together with changes in labile C fractions, aggregation, nutrient cycling or plant response. The Hawaiian and Chilean studies further show that SOC or SOM fractions should be interpreted jointly with mineralogy, land-use history and changes in physical or biological functions rather than through a single universal scoring curve [33,56,57,58,59].

Similar considerations apply to aggregate stability and active carbon. Results are strongly dependent on pretreatment, aggregate-size fraction, wetting procedure, sieving energy and the operational definition of the measured fraction. A value obtained using one aggregate-stability protocol should not be classified using a distribution developed with another method. Active-C and protein indices are likewise method-defined indicators whose benchmarks are valid only for the corresponding extraction and analytical procedure.

Available water capacity also requires a contextual reference basis. AWC should be defined as the difference in water content between explicitly specified upper and lower matric-potential limits, because the selected limits and the procedure used to determine the water-retention curve affect the resulting value. Texture-specific ranges may provide an initial comparison, but volcanic soils frequently display water-retention behavior that is not captured adequately by texture alone. Studies in Tenerife *sorriba* soils and irrigated Chilean Andosols confirm that plant-available water and hydraulic behavior depend on residual andic properties, pore structure, soil depth and system-specific calibration [15,17]. Mineralogy, organic matter, bulk density, aggregation, pore-size distribution and hysteresis can all modify the amount and accessibility of stored water. Furthermore, an apparently high AWC does not guarantee adequate water supply if rooting depth, hydraulic conductivity, aeration or irrigation frequency are limiting. AWC benchmarks should therefore be developed at the root-zone scale and related to crop water uptake and irrigation performance.

Reference ranges and peer-group benchmarks should thus be interpreted as comparative evidence. A high percentile indicates that a soil has a relatively high value within the specified population, not necessarily that it has reached a functional optimum. The definition of the peer group, analytical method and outcome against which the benchmark has been validated should be reported explicitly.

### 5.4. Indicators Requiring Relative or Temporal Interpretation

For several biological and functional indicators, the available evidence does not support direct classification into universally favorable or unfavorable ranges. These indicators may be highly sensitive to management and disturbance, but their values depend on short-term environmental and methodological conditions. Their main diagnostic strength lies in controlled comparisons among treatments, management systems, depths, reference sites or sampling dates.

Basal respiration and SIR are influenced by microbial biomass, availability of labile carbon and nutrients, moisture, temperature, aeration, recent plant inputs and sample pretreatment [20,21,22,23]. High respiration may indicate an active microbial community and rapid nutrient cycling, but it may also reflect disturbance-induced mineralization or inefficient loss of SOC. Conversely, low respiration may indicate low biological activity, but it can also occur in stable soils with limited readily decomposable substrate. Interpretation therefore requires complementary information on SOC, microbial biomass or SIR, moisture status and the management or disturbance context. In a Patagonian Andisol, microbial respiration and microbial biomass differed among native forest, pine plantation and degraded prairie and varied with depth, illustrating the value of respiration as a comparative land-use indicator rather than as a universal diagnostic class [62].

The metabolic quotient, qCO_2_, is often interpreted as an indicator of microbial C-use efficiency or stress [21]. Higher values may suggest greater respiratory loss per unit microbial biomass, but qCO_2_ also varies with community composition, substrate quality, nutrient limitation, succession, temperature and the methods used to determine respiration and biomass. It is consequently more defensible as a relative indicator measured under standardized conditions than as a variable with a universal critical value.

CLPP analysis characterizes the pattern of substrate-induced respiration rather than a single soil function [22,23]. Derived variables such as substrate richness, Shannon diversity, evenness or average metabolic response are highly dependent on the substrates selected, incubation conditions, data transformation, and treatment of low or negative responses. Their strongest use is therefore comparative: identifying shifts in potential functional profiles among treatments or over time. Absolute values should not be transferred among studies unless the experimental platform and data-processing procedure are equivalent.

Enzyme activities have a similar limitation. They can indicate potential capacities associated with C, N, P or S cycling, but measured activities depend on assay substrate, pH, temperature, incubation time, soil storage, and whether extracellular enzymes are stabilized on mineral or organic surfaces [24,25]. Ratios among enzyme activities may provide information on relative resource limitation, but they do not eliminate method dependence. Normalization by SOC, microbial biomass or another soil property can aid interpretation, although it may also introduce additional assumptions. A regional biological-quality index has also been proposed for volcanic Andisols and Aridisols of the Canary Islands using SOC, field and laboratory respiration, microbial biomass, hot-water-extractable C and enzyme activities [63]. The index discriminated among maturity and degradation states in natural ecosystems, demonstrating the feasibility of constructing regional biological references. However, it was not calibrated for agricultural soils and should not be transferred directly as an agronomic threshold.

AMF colonization, spore abundance and related plant–fungal measurements are functionally relevant but do not follow a simple “more is better” relationship [26,27,28]. Colonization may decline when P availability is high because the plant derives less benefit from the symbiosis, whereas high colonization may reflect either effective mutualism or strong nutrient limitation. Values also depend on host identity, phenology, root sampling, staining and counting procedure. AMF indicators should therefore be interpreted together with plant nutrition, biomass, root traits and soil P availability.

Measurements of glomalin-related soil proteins (GRSPs) require particular caution because the extracted fractions are operationally defined and do not represent a chemically pure or exclusively AMF-derived protein pool [29,30,31,32]. GRSP may correlate with aggregation, SOC or management, but it should be used as a comparative biochemical fraction rather than assigned a universal diagnostic threshold.

Infiltration and related hydraulic indicators are also context-sensitive. Measurements vary with the method used, initial water content, surface condition, antecedent rainfall or irrigation, water repellency, sealing, macropore connectivity and measurement scale. A low infiltration rate may signal compaction or surface sealing, but it can also reflect a naturally fine or highly water-retentive soil. Conversely, rapid infiltration may indicate favorable structure or preferential flow that bypasses much of the soil matrix. Functional interpretation requires integration with drainage, hydraulic conductivity, water retention, runoff, aeration and rooting conditions. Evidence from Tenerife demonstrates that the dominant control on infiltration differs among volcanic soil groups, whereas post-eruption monitoring shows that infiltration and aggregate stability may follow land-use-dependent recovery trajectories [16,18].

Where robust reference populations are unavailable, temporal monitoring can provide the most defensible interpretation. Repeated measurements within the same soil reduce, but do not eliminate, the influence of inherent soil properties. Sampling should be standardized by season, depth, soil moisture, crop stage and analytical procedure, and preferably combined with an untreated or locally representative reference. The magnitude of change should also be evaluated against analytical uncertainty and spatial variability. A statistically detectable change is not automatically functionally important; its relevance is strengthened when it persists over time and converges with independent soil or plant indicators [18].

### 5.5. Andic Diagnostic Criteria as Context Qualifiers

Andic diagnostic criteria occupy a different interpretive category from soil health thresholds. They are designed to identify a set of soil properties and pedogenic processes, not to distinguish healthy from degraded soil. Their role in soil health assessment is to define the context within which conventional indicators should be interpreted. Their presence should not be inferred solely from volcanic parent material or from recent ash deposition, which represents a disturbance context rather than evidence of pedogenically developed andic properties.

The World Reference Base for Soil Resources identifies andic properties using combinations of low bulk density, oxalate-extractable Al (Al_ox_) and Fe (Fe_ox_) and high phosphate retention [10]. For example, one diagnostic pathway combines bulk density ≤ 0.9 kg dm^−3^ with Al_ox_ + 0.5Fe_ox_ ≥ 2%. Additional measurements, including operationally defined oxalate- and pyrophosphate-extractable Al and Fe fractions, pH in NaF and independent mineralogical evidence, can help characterize the relative contribution of short-range-order phases and organo-metal associations and thereby clarify the mechanisms responsible for soil behavior.

These criteria should not be converted into soil health scores. Meeting a diagnostic criterion for andic properties does not imply that a soil is well managed, productive or free from degradation. Similarly, a higher concentration of oxalate-extractable Al or greater phosphate retention is not inherently better. These properties may support high aggregation, low bulk density, strong SOC stabilization and high water retention, while simultaneously contributing to low P availability, strong pH-dependent charge and complex nutrient interactions [7,8,9,10].

Bulk density illustrates the distinction particularly clearly. In many non-volcanic mineral soils, high bulk density can be evaluated against texture-based limits associated with root restriction. However, commonly used guidance from the USDA Natural Resources Conservation Service (NRCS) explicitly states that its general bulk-density limits do not apply to volcanic ash soils [60]. In volcanic soils, low bulk density may be an inherent consequence of andic materials rather than evidence of recent structural improvement. Diagnosis of compaction should therefore be based on deviation from a relevant volcanic-soil reference, changes through time, penetration resistance, porosity and root or hydraulic response rather than on generic mineral-soil limits.

Likewise, phosphorus retention; operationally extractable Al, Fe and Si fractions; mineralogical composition; specific surface area; surface-charge behavior; and microstructural or local elemental characteristics are primarily mechanistic or context-defining measurements. They help explain why routine indicators behave as they do and help distinguish intrinsic volcanic reactivity from management-induced effects. Their contribution to soil health assessment lies in reducing interpretive uncertainty, not in generating an independent score.

Andic criteria should therefore be used as context qualifiers, covariates or stratification variables when developing reference populations. They can identify soils for which generic thresholds are inappropriate and indicate when local calibration is needed. This distinction prevents intrinsic volcanic properties from being misclassified as either evidence of superior health or symptoms of degradation.

### 5.6. Evidence Gaps and Future Research Priorities for Volcanic Island Soils

The literature reveals a gradient rather than a binary distinction between indicators with and without interpretive support. Method-matched criteria for ECe, crop salinity response, selected pH constraints, calibrated nutrient tests, and some toxicity or regulatory endpoints can support relatively direct diagnosis. Method-defined soil-test P and other calibrated nutrient tests, SOC, bulk density, aggregate stability, water-related properties and microbial respiration have useful critical values or benchmark approaches, but these generally remain dependent on method, crop, region, soil group or reference population. qCO_2_, CLPP-derived indices, most enzyme activities, AMF measurements, GRSP fractions and advanced mechanistic indicators currently support mainly relative, temporal or explanatory interpretation.

Evidence relevant to the framework comes from both island and continental volcanic settings. Studies from continental volcanic soils, particularly in Chile and Patagonia, provide evidence on mechanisms, method suitability, and reference development associated with volcanic or andic properties, whereas studies from island settings, including Hawai’i and the Canary Islands, additionally capture island-specific agricultural, management and disturbance contexts [15,18,19,56,57,58,59]. However, these studies differ in climate, soil group, land use, analytical procedure and assessment objective and do not yet provide a transferable diagnostic system for volcanic island agriculture.

The principal gap is the shortage of outcome-validated criteria connecting standardized measurements with agronomic or environmental functions. Existing studies commonly demonstrate differences among land uses, treatments or dates, but rarely establish the magnitude of change associated with a meaningful change in crop performance, irrigation efficiency, nutrient-use efficiency, erosion resistance or recovery. Biological and hydrological indicators are particularly underdeveloped in this respect.

Future work should develop regional databases using harmonized sampling, pretreatment and analytical protocols. Reference populations should distinguish soil group, degree of andic expression, parent material or ash influence, moisture regime, depth, crop system and management history. Calibration should also distinguish agronomic critical values, environmental-risk thresholds and soil health benchmarks because these represent different outcomes and may imply different target ranges. In parallel, longitudinal designs should quantify analytical, spatial and seasonal variability and determine whether early biological responses predict longer-term changes in hydrological functioning, nutrient cycling or crop performance. External validation across contrasting volcanic islands should then establish which benchmark relationships are region-specific and which may be transferable among volcanic agroecosystems.

Until locally validated systems are available, interpretation should follow the evidence hierarchy described above, and the strength of evidence supporting each conclusion should be reflected in the diagnostic confidence assigned in Section 6.

## 6. Functional Integration and Diagnostic Confidence

Section 5 establishes the evidential basis available for interpreting individual indicators. The subsequent task is to integrate those results into a diagnosis without obscuring differences among soil functions or overstating the strength of the evidence. Integration should therefore be organized by the functional constraint under assessment and should distinguish direct diagnostic evidence from supporting and contextual evidence.

### 6.1. Integrating Convergent Evidence by Soil Function

Indicators should be integrated by soil function rather than by analytical discipline alone [5,6]. Recent comparisons of soil health assessment systems show that different scoring methods can produce low agreement and that aggregated indices may conceal trade-offs among functions [64]. A high overall score should therefore not compensate automatically for a severe limitation in salinity buffering, water regulation, nutrient availability or root-zone aeration.

The framework distinguishes among soil functions, mechanistic or contextual modifiers, and integrated functional outcomes. Soil indicators are therefore integrated primarily by the function under assessment, while volcanic-soil and andic properties provide explanatory context and plant or temporal responses provide independent functional validation. Each functional diagnosis should combine indicators that represent different components of the same process. Salinity risk, for example, may be evaluated through ECe, soluble-ion composition, ESP or SAR, irrigation-water quality, drainage and crop response. Water-regulation constraints may be evaluated through water retention, infiltration, saturated or unsaturated conductivity, aeration, effective rooting depth and plant-water response. Indicators of volcanic or andic reactivity provide contextual or mechanistic support when conventional measurements cannot distinguish inherent soil behavior from degradation.

Plant and biological indicators should be incorporated when they add independent evidence. Biological measurements may reveal early functional change, whereas plant biomass, nutrition, root development or physiology can establish whether the soil condition is expressed as an agronomic constraint. Their role is to strengthen or challenge the functional interpretation rather than create an additional uncontextualized score.

### 6.2. Grading Diagnostic Confidence and Resolving Conflicting Evidence

Diagnostic confidence should reflect both the quality of the interpretation criterion and the convergence of independent evidence.

The proposed high–moderate–limited classification is a qualitative expert framework intended to make diagnostic reasoning explicit and should not be interpreted as a validated scoring system. For operational use, confidence is evaluated along two dimensions: (i) the strength and local transferability of the interpretation basis and (ii) the degree of convergence among independent lines of evidence.

Interpretation-basis strength and transferability are considered strong when evidence is based on a method-matched, locally validated threshold or calibrated response relationship; intermediate when based on a relevant local reference or peer group, a persistent comparative or temporal relationship, or an external criterion with close methodological and contextual correspondence; and weak when based primarily on an externally derived benchmark without local validation or on mechanistic/contextual evidence not linked to a validated functional outcome. Evidence convergence is considered strong when multiple independent soil, site, plant or temporal lines of evidence agree; intermediate when support is partial or includes minor inconsistencies; and weak when the diagnosis relies on a single line of evidence or when independent indicators conflict. These two dimensions are combined in the qualitative decision matrix shown in Table 5.

Conflicting indicators should not be averaged into an apparently definitive score. They may indicate spatial or temporal heterogeneity, a mismatch among analytical procedures, compensating processes or an incorrectly specified functional hypothesis. Such cases should be reported as diagnostic uncertainty and used to identify the next measurement required—for example, plant response, effective CEC, root-zone hydraulic behavior or andic-reactivity indicators.

The final diagnosis should therefore identify the functional constraint, the evidence supporting it, the role of contextual or mechanistic indicators, the degree of convergence and the assigned level of confidence. This structure makes the reasoning traceable and prevents the distinction between intrinsic volcanic properties and management-induced change from being lost during integration.

## 7. Stepwise Workflow for Context-Sensitive Diagnosis

**Figure 2:**
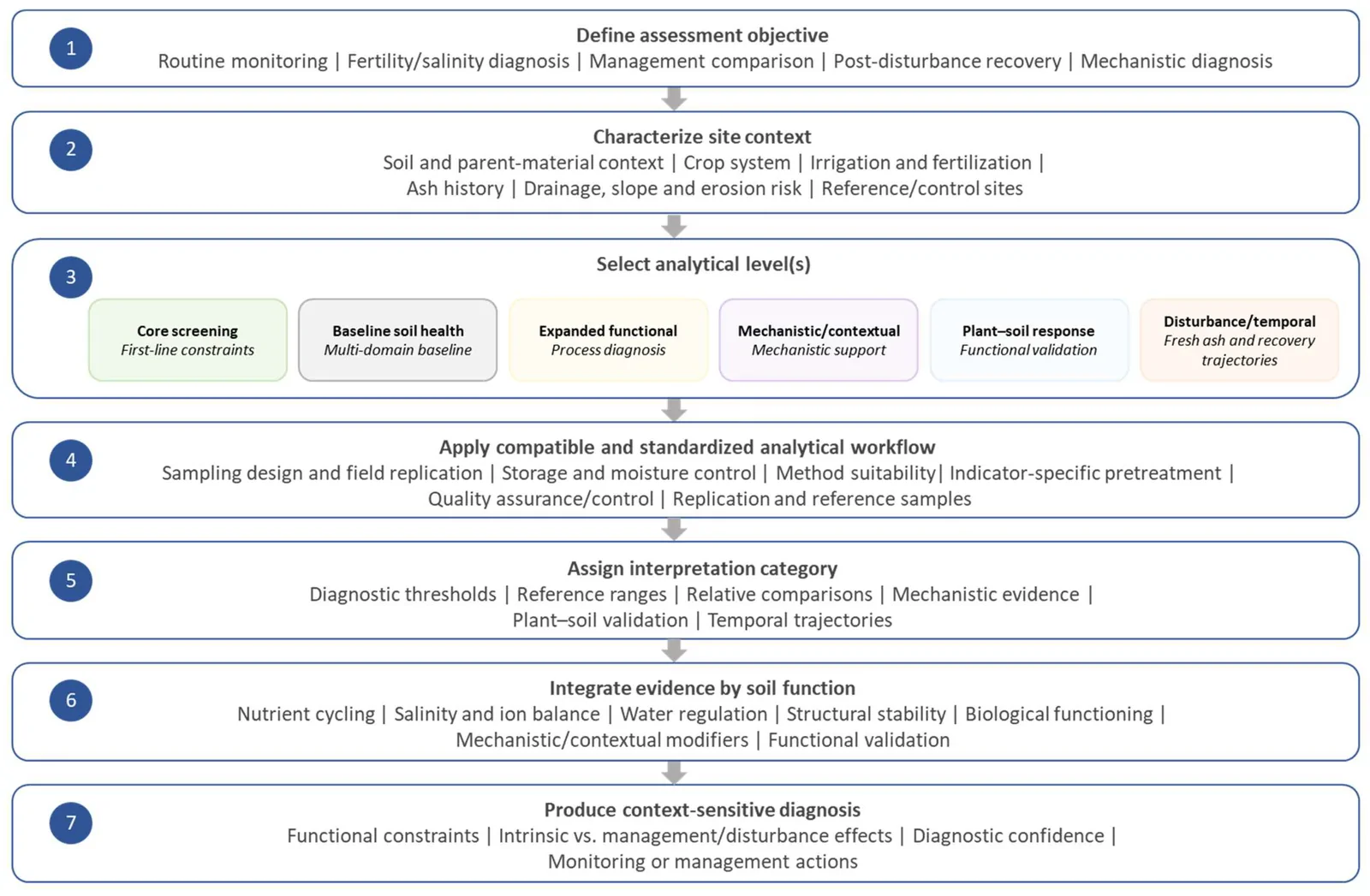
Stepwise workflow for context-sensitive soil health diagnosis in volcanic island agroecosystems. The workflow links the assessment objective and site context to fit-for-purpose analytical selection, compatible sampling and standardized analytical procedures, explicit interpretation bases, functional evidence integration and diagnosis with stated confidence.

The methodological components described above can be operationalized through the seven-step workflow shown in Figure 2. The workflow is intentionally flexible: analytical depth and supporting evidence should increase only when required by the assessment objective and remaining diagnostic uncertainty.

The first step is to define the assessment objective, because routine monitoring, fertility or salinity diagnosis, management comparison, volcanic-disturbance assessment, recovery monitoring and mechanistic investigation require different analytical and interpretive approaches.

The second step is to characterize the site context, including relevant soil and parent-material characteristics, crop and management system, irrigation and fertilization regime, disturbance history, drainage and erosion conditions, and the availability of suitable reference or control sites. This information defines the context for subsequent interpretation.

The third step is to select the appropriate analytical level. A core routine screening dataset may be sufficient for first-line identification of major constraints, whereas a baseline soil health dataset provides a broader characterization across chemical, physical and biological domains. Expanded functional, mechanistic/contextual, plant-response or disturbance/temporal measurements should be added when the initial evidence does not adequately resolve the diagnostic question.

The fourth step is to apply a compatible and standardized sampling and analytical workflow. Sampling design, including depth, timing and field replication, sample conservation, pretreatment and quality assurance and quality control should be appropriate to the property being measured, and procedures susceptible to volcanic-soil effects should be verified or explicitly qualified as described in Section 4.6 and Table 3.

The fifth step is to define the interpretation basis before integration. Each result should be identified as supporting a direct diagnostic threshold, reference range, relative comparison, mechanistic interpretation, plant validation or temporal trajectory, according to the strength and transferability of the evidence.

The sixth step is to integrate convergent evidence by soil function. Nutrient cycling, salinity and ion balance, water regulation, structural stability and biological functioning should be evaluated together with relevant mechanistic or contextual modifiers, while plant and temporal responses provide independent functional validation.

The final step is to produce a context-sensitive diagnosis that identifies the main functional constraints, distinguishes intrinsic soil properties from management- or disturbance-related effects, states the interpretation basis supporting each conclusion and assigns an explicit level of diagnostic confidence.

The workflow thus provides a traceable sequence from assessment objective to monitoring or management recommendations without requiring a universal indicator set or scoring system.

## 8. Practical Recommendations and Reporting Requirements

Practical application of the framework requires sufficient reporting of sampling design, analytical procedures, contextual information and diagnostic confidence to make the assessment reproducible and traceable. This emphasis on monitoring, transparent reporting and diagnostic traceability is consistent with the policy direction of the European Union Soil Monitoring Law, which establishes a common framework for soil monitoring and resilience while recognizing the need to assess soil condition and support soil health across Member States [65].

### 8.1. Fit-for-Purpose Indicator Selection

Reports should identify the assessment objective, targeted soil functions and selected analytical level, including whether the assessment is intended as core screening or as a broader baseline soil health characterization, and provide the rationale for adding expanded functional, mechanistic/contextual, plant-response or disturbance/temporal measurements. The selected dataset should be sufficient to support the intended diagnosis and reduce the relevant uncertainty, but it should not be exhaustive by default.

### 8.2. Sampling Design and Minimum Contextual Information to Report

Sampling design should be defined according to the assessment objective and the expected spatial and temporal heterogeneity before analytical results are interpreted. Sampling depth and horizon selection should represent the soil function or crop response under assessment, with root-zone sampling used where the diagnostic question concerns plant-available nutrients, salinity, water supply or rooting constraints. The use of individual or composite samples should be stated explicitly: composite sampling can provide a representative mean for relatively homogeneous management units, whereas individual field replicates are required when spatial variability, treatment effects or statistical inference are themselves part of the assessment.

A context-sensitive diagnosis is only reproducible if site and management information are reported together with analytical results. At minimum, assessments should describe soil type or parent material, degree of volcanic or andic influence, sampling depth and date, crop system, irrigation regime, fertilization and amendment history, drainage conditions and recent disturbance history. Where relevant, ash deposition, ash incorporation or ash removal should be documented, including timing, thickness or intensity, management intervention, and visible effects on the soil surface or root zone [13,14].

Sampling units should be stratified where relevant according to topography, soil or parent-material variation, management zones, irrigation patterns or disturbance intensity, rather than combining clearly heterogeneous areas into a single sample. Repeated assessments should use comparable locations, depths, crop stages, and seasonal or soil-moisture conditions whenever possible. Reference or control sites should be selected to represent an appropriate local comparison and sampled using the same design as the assessed sites. In post-eruption settings, freshly deposited ash, the underlying pre-existing soil and mixed ash–soil horizons should be sampled and reported separately where they remain distinguishable, because they represent different materials and stages of disturbance and pedogenic evolution.

The basis for comparison should also be stated explicitly. If interpretation relies on diagnostic thresholds, the source and local relevance of these thresholds should be indicated. If interpretation is based on reference ranges, the reference population should be described. For paired comparisons, management gradients or temporal trajectories, the factors being compared and the principal sources of variability controlled should be stated explicitly.

Analytical methods should be reported with enough detail to allow comparison, including sample pretreatment, moisture standardization, extraction, digestion or incubation conditions, analytical units, replication, and the quality assurance and quality control procedures applied. For fresh-ash leachates, the ash-to-water ratio, contact time, extraction sequence and filtration procedure should also be reported. The sample condition at analysis should be stated explicitly, including whether field-moist, air-dried, oven-dried or freeze-dried material was used, together with the drying temperature, duration, storage conditions and any rewetting procedure. Reports should also indicate whether procedures requiring adaptation in volcanic soils were used; how method suitability was verified; and whether the reported value represents a primary-particle distribution, a field-state property, a potential property, an operationally defined fraction or, for elemental analysis, a total or pseudo-total concentration. Operationally defined fractions, such as glomalin-related soil proteins, should be reported as such rather than as direct measurements of a single biological entity [29,30,31,32]. Hydrological indicators should specify the measured domain and analytical configuration, including whether water-retention measurements were obtained on intact or minimally disturbed cores or on repacked material and whether samples were maintained field-moist or were dried and rewetted. WHC should be reported as an operational measurement together with the procedure used, whereas AWC should include the upper and lower matric-potential limits used for its calculation.

### 8.3. Minimum Analytical Quality Assurance and Quality Control Requirements

Analytical results used for soil health diagnosis should be supported by a fit-for-purpose quality assurance and quality control scheme appropriate to the measurand and analytical procedure. At minimum, this should include laboratory duplicates or replicate determinations, where appropriate; reagent or method blanks; calibration verification; and suitable reference or certified reference materials, when available. Extraction-based procedures should include appropriate extraction or recovery controls, particularly where matrix effects are expected, and results reported on a dry-mass basis should include a documented moisture correction.

For measurements conducted in multiple analytical batches, a consistent control or reference sample should be included to detect inter-batch drift and support comparability over time. Detection and quantification limits should be established and reported when they affect interpretation, particularly for concentrations close to diagnostic, agronomic or regulatory criteria. Field duplicates can additionally be used to distinguish sampling variability from analytical variability. For solution or extract chemistry, internal consistency checks should be applied where chemically appropriate and sufficient analytes are available. Analytical uncertainty should be reported or otherwise characterized at a level appropriate to the intended diagnosis, so that small differences are not interpreted as meaningful changes when they fall within expected analytical variability.

### 8.4. Reporting Diagnostic Confidence and Uncertainty

Final reports should make the diagnostic reasoning traceable. For each main conclusion, authors should identify the soil function assessed, the indicators supporting the conclusion, the interpretation basis used and whether the evidence was direct, supporting or contextual. Any conflicting evidence should be reported rather than concealed within an aggregate score.

Reports should assign a qualitative level of diagnostic confidence—high, moderate or limited—using the criteria described in Section 6.2 and Table 5. The interpretation-basis strength and local transferability, the degree of convergence among independent lines of evidence and the reasoning supporting the assigned confidence level should be stated explicitly. The classification should be reported as a qualitative expert judgment rather than as a validated numerical score. Reports should also identify the main sources of uncertainty, including sampling representativeness, analytical compatibility, spatial variability, seasonality, absence of local calibration or incomplete agreement among soil and plant indicators.

## 9. Conclusions

Soil health assessment in volcanic island agroecosystems requires explicit separation of indicator selection, analytical measurement, interpretation and functional integration. Volcanic-soil properties and, where expressed, andic properties can modify not only the expected values of conventional physical, chemical and biological indicators, but also the validity of sample pretreatments and analytical procedures developed for other mineral soils. The framework proposed here therefore combines fit-for-purpose analytical levels with method-suitability checks, explicit interpretation bases and integration by soil function, while distinguishing mechanistic or contextual modifiers from functional outcomes and making diagnostic confidence explicit.

The reviewed evidence shows a clear gradient of diagnostic support. Some agronomic constraints can be evaluated using method-matched thresholds or crop-response relationships, whereas many dynamic properties require volcanic-soil reference populations, controlled comparisons or temporal monitoring. Regional initiatives in Chile, Hawai’i and the Canary Islands demonstrate that context-adapted indicator sets, benchmarks and biological or functional assessment systems can be developed. However, many physical, biological and functional indicators still lack locally validated reference populations or outcome-based criteria for volcanic island agriculture, and the transferability of existing regional approaches remains limited by differences in mineralogy, degree of andic expression, climate, crop system, management history, sampling design and analytical procedure.

By integrating indicators by soil function and reporting the basis and confidence of each conclusion, the proposed approach avoids treating intrinsic volcanic properties as direct evidence of either improvement or degradation. Three research priorities follow from these limitations. First, regional reference databases should be developed using harmonized sampling and analytical protocols and stratified by relevant soil, crop, management and disturbance contexts. Second, indicator–function relationships and diagnostic criteria should be externally validated across contrasting volcanic islands and linked to measurable crop, hydrological, biological and environmental outcomes. Third, long-term monitoring datasets should be strengthened to quantify management and post-disturbance trajectories while separating persistent functional change from spatial, seasonal and analytical variability. Progress in these areas would provide a transparent basis for monitoring and management recommendations and support the development of locally validated and increasingly transferable diagnostic systems for volcanic island agriculture.

## Figures and Tables

**Table 1:** Interpretation categories for soil health indicators in volcanic island agroecosystems.

Interpretation Category	Representative Properties or Measurements	Main Use in Volcanic Island Agroecosystems
Diagnostic-threshold indicators	pH, EC, exchangeable Na, SAR/ESP, soluble Cl, selected nutrient concentrations, potentially toxic element concentrations	First-level diagnosis of salinity, sodicity, pH constraints, nutrient deficiency or toxicity, using locally valid thresholds
Reference-range indicators	Soil-test P (extraction method specified), exchangeable cations, CEC, SOC, total N, bulk density, aggregate stability, AWC	Benchmarking among comparable soils, crops, depths or management systems
Relative functional indicators	Basal respiration, SIR, qCO_2_, CLPP-derived responses, enzyme activities, AMF colonization or spore abundance, GRSP fractions, infiltration	Detection of functional changes across treatments, gradients, sites or time points
Mechanistic/contextual indicators	Phosphate retention, NaF reactivity, operationally extractable Al, Fe and Si fractions, mineralogical and bulk elemental composition, specific surface area, surface-charge behavior, microstructural features, local elemental associations	Mechanistic or contextual interpretation of phosphate retention, C stabilization, aggregation, variable charge, surface reactivity and volcanic-material influence
Integrated plant–soil response indicators	Biomass, root traits, foliar nutrient concentrations, leaf chlorophyll status/SPAD readings, gas exchange, water potential, AMF response	Validation of whether soil changes translate into crop or physiological responses
Temporal recovery indicators	Repeated measurements of selected soil, biological and plant variables	Monitoring degradation, adaptation or recovery after ash deposition, ash incorporation or restoration

Categories are not mutually exclusive. The same indicator may shift category depending on the assessment objective, crop system, volcanic soil properties, management history and disturbance or recovery context. Abbreviations: AMF, arbuscular mycorrhizal fungi; AWC, available water capacity; CEC, cation exchange capacity; CLPP, community-level physiological profiles; EC, electrical conductivity; ESP, exchangeable sodium percentage; GRSP, glomalin-related soil proteins; SAR, sodium adsorption ratio; SIR, substrate-induced respiration; SOC, soil organic carbon; SPAD, Soil Plant Analysis Development; qCO_2_, metabolic quotient.

**Table 2:** Analytical levels for context-sensitive soil health assessment in volcanic island agroecosystems.

Analytical Level	Core Purpose	Typical Assessment Contexts
Core routine screening dataset	First-line identification of major agronomic constraints using broadly accessible measurements	Rapid screening; routine fertility or salinity assessment; laboratories with limited analytical capacity
Baseline soil health dataset	Broader multi-domain characterization of chemical, physical and biological soil condition	Baseline characterization; routine monitoring; farm, plot or management comparisons
Expanded functional dataset	Assessment of hydrological behavior, biological functioning and early functional change	Management effects; early degradation; biological recovery; unexplained soil or crop responses
Mechanistic/contextual dataset	Explanation of mineralogical, chemical and surface-reactivity controls	Ambiguous P availability; high SOC stabilization; variable charge; active Al/Fe phases; ash effects
Integrated plant–soil response dataset	Validation of soil constraints through crop growth, nutrition or physiology	Crop-focused diagnosis; salinity or water stress; amendment trials; ash or substrate effects
Disturbance and temporal monitoring dataset	Characterization of fresh volcanic disturbance and assessment of adaptation or recovery trajectories	Fresh ash and ash-leachate characterization; ash-affected soils; post-eruption recovery; restoration; long-term monitoring

Analytical levels are cumulative and flexible. Core screening is intended to identify major first-line constraints and does not constitute a complete soil health characterization; the baseline dataset extends the assessment across chemical, physical and biological domains. Additional levels should be selected according to the assessment objective, diagnostic uncertainty and site context. Abbreviation: SOC, soil organic carbon.

**Table 4:** Representative published interpretation bases for selected soil health indicators and regional assessment approaches, and their applicability to volcanic island agroecosystems.

Indicator and Analytical Basis	Interpretation Evidence	Transferability and Key Qualification	Representative Sources
Salinity and sodicity: ECe, ESP and SAR	Direct diagnostic criteria: historical ECe boundary ≈4 dS m^−1^ and ESP ≈15%; crop-specific ECe threshold–slope models	Useful when method, matrix and crop context match. ECe criteria cannot be transferred directly to EC measured in diluted suspensions; ESP and SAR remain operational and context dependent	[45,48]
Soil pH	Direct or crop-specific diagnostic ranges linked to acidity, alkalinity, nutrient limitation or toxicity	Specify crop and pH method. In variable-charge volcanic soils, pH also acts as a mechanistic control of CEC, P retention, Al activity and surface charge	[5,6,7,8,9,10]
Olsen-extractable P	Crop-, method- and region-specific critical value; ≈33 mg P kg^−1^ reported for potato in Southern Chile	Demonstrates that crop-calibrated criteria can be developed, but the value is not universal and is restricted to the Olsen method, crop, depth, region and calibration model	[49]
Other calibrated nutrient tests	Crop-, method- and region-specific sufficiency classes or critical values	Apply only with the same or demonstrably equivalent extraction procedure and an appropriate crop-response calibration	[50,51,52]
Specific-ion toxicity (B, Cl and Na)	Crop-specific response criteria for irrigation water, soil solution or saturation extracts	Matrix, crop/cultivar, irrigation method and soil-water regime must match; water and soil-extract concentrations are not interchangeable	[53]
Potentially toxic trace element concentrations	Regulatory criteria and method-specific assessment of mobility/bioavailability	Match jurisdiction, receptor, measured fraction and digestion/extraction procedure; total, pseudo-total, extractable and soil-solution concentrations are not interchangeable	[46,47]
SOC and labile or operational SOM fractions	Reference distributions, peer-group benchmarks and regionally adjusted scoring approaches	Appropriate when reference populations account for volcanic mineralogy, moisture regime, soil group and land-use history. High SOC may reflect intrinsic andic stabilization rather than recent management, and operational SOM fractions remain method-specific	[33,54,55,56,57,58,59]
Structural indicators: bulk density and wet aggregate stability	Texture- or soil-specific benchmarks, peer-group distributions and relative or temporal comparisons	Generic mineral-soil limits may not apply to volcanic soils. Bulk density should be compared with relevant volcanic-soil references, while aggregate-stability values are strongly dependent on pretreatment, aggregate size, wetting procedure and analytical protocol	[10,15,18,19,33,54,55,60]
Hydrological indicators: AWC, infiltration and hydraulic properties	Contextual reference ranges and relative or temporal comparisons	Interpretation should account for volcanic mineralogy, pore structure, root-zone characteristics and measurement method. For AWC, the upper and lower matric-potential limits and the sample configuration used to determine water retention should be reported. High water retention does not necessarily imply high plant-available water, and infiltration is strongly affected by antecedent moisture, surface condition, preferential flow and measurement scale	[7,8,15,16,17,18,19,54,61]
Microbial and functional indicators: respiration, qCO_2_, CLPP and enzyme activities	Peer-group benchmarks where available; otherwise, relative or temporal comparison under standardized conditions	No generally validated universal thresholds are available. Results depend strongly on moisture, temperature, substrate availability, incubation or assay conditions, sample storage, microbial biomass and data-processing procedures	[20,21,22,23,24,25,55,56,62]
AMF and GRSP-related measurements	Relative, plant-supported or temporal interpretation in relation to host response, P status, management or disturbance	Higher values are not necessarily better. AMF responses depend strongly on host identity and phenology, while GRSP fractions are operationally defined and should not be interpreted as direct measurements of a unique AMF-derived compound	[26,27,28,29,30,31,32]
Andic diagnostic criteria	Soil-classification criteria and context qualification based on WRB diagnostic properties	Directly useful for identifying andic properties and defining the appropriate interpretation context, but these criteria describe pedogenic properties rather than soil health and should not be converted into health scores	[10]
Mechanistic/context-defining soil properties	Mineralogical composition, specific surface area, surface-charge behavior, microstructural and local elemental characteristics, and operationally extractable Al–Fe–Si fractions, characterized using appropriate method-specific analytical procedures	Useful when routine indicators are ambiguous or strongly controlled by volcanic materials. Selectively extractable Al, Fe and Si fractions are operationally defined and should not be interpreted as unique mineralogical or chemical pools. Likewise, XRD primarily provides evidence of crystalline mineral phases, whereas SEM–EDS provides spatially local microstructural and elemental information rather than representative bulk composition. These measurements do not provide direct soil health thresholds and must be integrated with conventional indicators and functional or plant responses	[7,8,9,10]
Regionally adapted multi-indicator soil health assessment	Regional reference distributions, scoring functions or integrated assessment frameworks adjusted for inherent soil and land-use factors	Provides a model for developing volcanic-island reference systems, but existing frameworks remain regionally bounded and require validation before transfer among archipelagos, climates or contrasting volcanic-soil groups	[19,56,57,58]

The table provides representative examples and is not intended as an exhaustive compilation of agronomic or regulatory criteria. Numerical values should only be applied when the analytical method, units, measured soil fraction, crop or environmental receptor, and relevant soil context correspond to those used to derive the criterion. Interpretation evidence and transferability should therefore be considered jointly rather than treating published values as universally applicable. Abbreviations: AMF, arbuscular mycorrhizal fungi; AWC, available water capacity; CEC, cation exchange capacity; CLPP, community-level physiological profiles; EC, electrical conductivity; ECe, electrical conductivity of the saturated-paste extract; ESP, exchangeable sodium percentage; GRSP, glomalin-related soil proteins; SAR, sodium adsorption ratio; SOC, soil organic carbon; SOM, soil organic matter; qCO_2_, metabolic quotient.

**Table 5:** Qualitative matrix for assigning diagnostic confidence from interpretation-basis strength and evidence convergence.

Interpretation-Basis Strength and Local Transferability	Strong Convergence	Intermediate Convergence	Weak or Conflicting Convergence
Strong	High	Moderate	Limited
Intermediate	Moderate	Moderate	Limited
Weak	Moderate	Limited	Limited

The matrix is a proposed qualitative decision aid rather than a validated numerical scoring system. High confidence requires both a strong, locally transferable interpretation basis and strong convergence among independent lines of evidence. Strong convergence can increase confidence when the interpretation basis is weaker, but cannot by itself support a high-confidence diagnosis.

## Data Availability

No new experimental data were generated in this study. The literature sources used to support the review are cited in the reference list.

## Supplementary Materials

The following supporting information can be downloaded at https://www.mdpi.com/article/10.3390/mps9050129/s1, Table S1: Targeted literature-search strategy used to support the critical methodological review.

## Abbreviations

The following abbreviations are used in this manuscript:

AMF	Arbuscular mycorrhizal fungi
AWC	Available water capacity
CEC	Cation exchange capacity
CLPP	Community-level physiological profiles
DTPA	Diethylenetriaminepentaacetic acid
EC	Electrical conductivity
ECe	Electrical conductivity of the saturated-paste extract
ESP	Exchangeable sodium percentage
GRSP	Glomalin-related soil proteins
ISO	International Organization for Standardization
LOI	Loss on ignition
NH_4_OAc	Ammonium acetate
NRCS	Natural Resources Conservation Service
SAR	Sodium adsorption ratio
SEM-EDS	Scanning electron microscopy with energy-dispersive X-ray spectroscopy
SHAPE	Soil Health Assessment Protocol and Evaluation
SIR	Substrate-induced respiration
SOC	Soil organic carbon
SOM	Soil organic matter
SPAD	Soil Plant Analysis Development
TN	Total nitrogen
USDA	United States Department of Agriculture
WHC	Water-holding capacity
WRB	World Reference Base for Soil Resources
XRD	X-ray diffraction
qCO_2_	Metabolic quotient
