# Supplementary material for: An Integrated Methodological Framework for SoilHealth Assessment in Volcanic Island Agroecosystems: Indicator Selection, Analytical Workflows and Context-Sensitive Interpretation
Source: Methods Protoc. 2026 Sep 1;9(5):129. doi: 10.3390/mps9050129 (PMC13600072; doi:10.3390/mps9050129)
Supplement: Supplementary file 1 [file mps-09-00129-s001.zip › mps-4499800-supplementary.pdf]

Table S1. Targeted literature-search strategy used to support the critical methodological review.

Search domain	Web of Science Core Collection search string	Scopus search string	WoS: Records retrieved	Scopus: Records retrieved
1. Soil-health frameworks and indicator interpretation	TS=(("soil health" OR "soil quality") AND<br>(assessment OR framework* OR indicator* OR benchmark* OR threshold* OR<br>"critical value*" OR "reference range*" OR "peer group*" OR scoring) AND<br>(agricultur* OR agroecosystem*))	TITLE-ABS-KEY(<br>("soil health" OR "soil quality") AND<br>(assessment OR framework* OR indicator* OR benchmark* OR threshold* OR<br>"critical value*" OR "reference range*" OR "peer group*" OR scoring) AND<br>(agricultur* OR agroecosystem*)<br>)	5,979	7,585
2. Volcanic/andic soils and soil-health assessment	TS=((Andosol* OR Andisol* OR "volcanic soil*" OR "volcanic ash soil*" OR<br>"andic propert*") AND<br>("soil health" OR "soil quality" OR indicator* OR assessment OR benchmark*<br>OR "reference range*" OR management))	TITLE-ABS-KEY(<br>(Andosol* OR Andisol* OR "volcanic soil*" OR "volcanic ash soil*" OR<br>"andic propert*") AND<br>("soil health" OR "soil quality" OR indicator* OR assessment OR benchmark*<br>OR "reference range*" OR management)<br>)	1,020	1,129
3. Chemical and mineralogical method suitability	TS=((Andosol* OR Andisol* OR "volcanic soil*" OR "volcanic ash soil*" OR<br>"andic propert*") AND<br>("cation exchange capacity" OR "phosphate retention" OR "soil test phosphorus"<br>OR "available phosphorus" OR oxalate OR pyrophosphate OR dithionite OR<br>"selective extraction*" OR "loss on ignition" OR "dry combustion" OR<br>mineralog* OR allophane OR imogolite OR ferrihydrite))	TITLE-ABS-KEY(<br>(Andosol* OR Andisol* OR "volcanic soil*" OR "volcanic ash soil*" OR<br>"andic propert*") AND<br>("cation exchange capacity" OR "phosphate retention" OR "soil test phosphorus"<br>OR "available phosphorus" OR oxalate OR pyrophosphate OR dithionite OR<br>"selective extraction*" OR "loss on ignition" OR "dry combustion" OR	1,011	1,039

Search domain	Web of Science Core Collection search string	Scopus search string	WoS: Records retrieved	Scopus: Records retrieved
		mineralog* OR allophane OR imogolite OR ferrihydrite))		
4. Physical and hydrological method suitability	TS=((Andosol* OR Andisol* OR "volcanic soil*" OR "volcanic ash soil*" OR "andic propert*") AND ("particle size" OR granulometr* OR dispers* OR drying OR rewetting OR "bulk density" OR "aggregate stability" OR "water retention" OR "available water capacity" OR infiltration OR hydraulic*))	TITLE-ABS-KEY((Andosol* OR Andisol* OR "volcanic soil*" OR "volcanic ash soil*" OR "andic propert*") AND ("particle size" OR granulometr* OR dispers* OR drying OR rewetting OR "bulk density" OR "aggregate stability" OR "water retention" OR "available water capacity" OR infiltration OR hydraulic*))	1,150	838
5. Biological and functional indicators	TS=((Andosol* OR Andisol* OR "volcanic soil*" OR "volcanic ash soil*") AND (respiration OR "microbial biomass" OR qCO2 OR "metabolic quotient" OR "community-level physiological profile*" OR enzyme* OR mycorrhiz* OR glomalin OR "biological indicator*"))	TITLE-ABS-KEY((Andosol* OR Andisol* OR "volcanic soil*" OR "volcanic ash soil*") AND (respiration OR "microbial biomass" OR qCO2 OR "metabolic quotient" OR "community-level physiological profile*" OR enzyme* OR mycorrhiz* OR glomalin OR "biological indicator*"))	532	496
6. Fresh volcanic ash, disturbance and recovery	TS=(("volcanic ash" OR tephra) AND (agricultur* OR soil* OR crop* OR agroecosystem*) AND (leachat* OR "soluble ion*" OR salinity OR phytotox* OR incorporation OR	TITLE-ABS-KEY(("volcanic ash" OR tephra) AND (agricultur* OR soil* OR crop* OR agroecosystem*) AND	702	602

Search domain	Web of Science Core Collection search string	Scopus search string	WoS: Records retrieved	Scopus: Records retrieved
	recovery OR weathering OR "soil function*" OR monitoring))	(leachat* OR "soluble ion*" OR salinity OR phytotox* OR incorporation OR recovery OR weathering OR "soil function*" OR monitoring))		
7. Island agricultural context	TS=((island* OR archipelago*) AND (agricultur* OR agroecosystem*) AND (soil* OR "soil health") AND (irrigation OR salinity OR management OR volcanic OR disturbance))	TITLE-ABS-KEY((island* OR archipelago*) AND (agricultur* OR agroecosystem*) AND (soil* OR "soil health") AND (irrigation OR salinity OR management OR volcanic OR disturbance))	855	1,134

Searches were conducted without a lower publication-year restriction and were last updated on 21 August 2026. The document-type restriction retained peer-reviewed Articles and Reviews. “Records retrieved” indicates the number returned by the database search before document-type restriction. Searches were targeted rather than exhaustive and were used to identify and update methodologically and interpretively relevant evidence for the critical methodological review.
